# Dual Medical Therapy for Treatment of Arrhythmias in Cardiac Sarcoidosis

**DOI:** 10.19102/icrm.2022.13113

**Published:** 2022-11-15

**Authors:** Robert Sibilia, Robert Baughman, Daniel Washko, Elyse Lower, Alexandru Costea

**Affiliations:** ^1^Department of Internal Medicine, University of Cincinnati Medical Center, Cincinnati, OH, USA; ^2^Department of Cardiology, Mercy Health, Fairfield, OH, USA

**Keywords:** Anti-arrhythmics, cardiac devices, immunosuppression, sarcoidosis, ventricular arrhythmias

## Abstract

Anti-arrhythmics can be useful for ventricular arrhythmias in cardiac sarcoidosis (CS) that are refractory to immunosuppression. However, there is conflicting evidence on the efficacy of immunosuppression for treating arrhythmias in CS patients and a lack of data to support using immunosuppression alone as an initial strategy. The objective of this study was to assess for differences in arrhythmia burden over time with currently used immunosuppression and anti-arrhythmic regimens. Patients with CS and implanted cardiac devices were identified from a single-center registry. Study participants were retrospectively classified based on the medication regimen as follows: control (no therapy), immunosuppression, anti-arrhythmics, or dual therapy. Device interrogations were reviewed for premature ventricular contractions (PVCs), non-sustained ventricular tachycardia (NSVT), and device firings over time. Interrogations for 42 patients were reviewed over a mean period of 31 months. Regression analysis showed a significant decrease in the frequencies of PVCs (slope, −1.47; *P* = .04) and NSVT (slope, −0.05; *P* = .01) for patients on dual therapy compared to an increase or no change in the other groups. Across all patients, there was no difference between groups in the percentage of patients experiencing device firings. In a subset analysis of patients with implantable cardioverter-defibrillators for primary prevention, 6% on dual therapy required device firings compared to 43% and 40% on single or no therapy, respectively (*P* = .049, χ^2^ = 6.02). In conclusion, patients on both immunosuppression and anti-arrhythmics had a reduction in PVCs and NSVT over time. Overall, there were no differences between groups in terms of device firings, except in a subset analysis of patients with no history of ventricular tachycardia.

## Introduction

Cardiac sarcoidosis (CS) is associated with a poor prognosis, with patients having an estimated 5-year survival rate of 60%–75%.^1,2^ It is the cause of 13%–25% of deaths from sarcoidosis in the United States and up to 85% of deaths in Japan.^1–3^ The most common causes of death in this population are progressive heart failure, sustained ventricular arrhythmias, or complete heart block.^2^ The rates of sudden cardiac death in CS have been reported as 24%–67% in older studies^4,5^; however, these have likely improved over time with advancements in imaging and treatment. Sustained ventricular arrhythmias are a leading cause of death and have been reported to occur in anywhere from 2%–42% of CS patients.^5^

There is currently no standardized approach for the medical management of CS.^6^ According to expert consensus, corticosteroids are used to control active inflammation in most patients.^7^ These drugs have been shown to stop the progression of cardiac fibrosis, improve left ventricular ejection fraction, restore the conduction system, and potentially reduce mortality.^1,2,8^ The effectiveness of corticosteroids for treating arrhythmias is unclear. Some studies have found a reduction in rates of ventricular tachycardia (VT) and ventricular fibrillation (VF), while others have shown no improvement or even a possible worsening of the patient’s condition.^9–12^ As corticosteroid therapy carries significant risks over time, non-steroidal immunosuppression has become increasingly used.^6,13^ These regimens are typically composed of an antiproliferative or biologic agent with or without low-dose steroids. Methotrexate with low-dose prednisone, for example, has been shown to stabilize cardiac function with fewer side effects than corticosteroids alone.^14^ While evidence for these regimens in the setting of arrhythmias is limited, they have shown promise when used long term without interruption.^9^ No prior studies have examined the impact of corticosteroid-sparing regimens on arrhythmias in the setting of concurrent anti-arrhythmic therapy.

The current guidelines for the use of anti-arrhythmics in CS are that they can be useful for arrhythmias that are refractory to immunosuppression (class IIa indication).^15^ However, data are lacking to support starting with immunosuppression alone, and there is no evidence to guide the selection of an anti-arrhythmic.^15^ Previous retrospective studies have reported effective management of ventricular arrhythmias using corticosteroids and anti-arrhythmics together based on 6-month device interrogations and Holter monitors.^16,17^ One prospective study on CS patients with VT/VF found that 50% experienced arrhythmia recurrence despite corticosteroids and required the addition of an anti-arrhythmic.^11^

In this context, our goals were to expand on previous studies and assess the effect of corticosteroid-sparing therapy both with and without anti-arrhythmics. We wanted to compare the effects of various medication regimens, provide information on the real-time impact of medical therapy, and report our experiences from a large sarcoidosis center.

## Methods

### Study population

The study included patients at the University of Cincinnati Sarcoidosis and Interstitial Lung Disease Clinic who had cardiac devices implanted from 2009–2019. Inclusion criteria were highly probable or probable CS based on the World Association of Sarcoidosis and Other Granulomatous Diseases criteria,^18^ an implanted cardiac device, and consistent device interrogations over time. Patients with loop recorders, interrogations at outside hospitals, or non-consistent medication regimens were excluded. Patients with pacemakers were excluded from the analysis of device firings (defined as anti-tachycardia pacing [ATP] or shock). This study was approved by the University of Cincinnati Medical Center Institutional Review Board and was registered at ClinicalTrials.gov (NCT02356445). Written informed consent was not required. The data collected for this study are available from the corresponding author upon request.

### Device indications and interrogation data

The indications for device placement included complete heart block, VT/VF, heart failure with reduced ejection fraction, frequent ectopy with syncope, inducible VT/VF, and a family history of sudden cardiac death in the setting of CS. Reduced ejection fraction was defined as an ejection fraction of ≤40%.^19^ Sudden cardiac death was defined as unexpected death thought to be due to a cardiac arrhythmia.^20^ Data from device interrogations were recorded starting at the time of device placement and ending 4 years afterward. All available interrogations in the patients’ charts were reviewed unless there were multiple interrogations in a month, in which case the first interrogation in that month was used. Incorrect arrhythmia classification or inappropriate device therapies were excluded from the results. All device firing events defined as ATP or shock were independently reviewed by 2 electrophysiologists.

### Arrhythmia definitions

Premature ventricular contractions (PVCs) were defined according to the American College of Cardiology/American Heart Association (AHA) as “a depolarization of the ventricle which occurs with a coupling interval shorter than that resulting from the intrinsic heart rhythm.”^21^ The PVC counts for patients were recorded as PVCs per hour based on the format of most interrogation reports. Non-sustained VT (NSVT) was defined as “≥3 consecutive complexes in duration emanating from the ventricles at a rate [of] >100 bpm” and lasting <30 s.^21^ NSVT events were recorded as a monthly average to correct for differences in interrogation frequency. VT was defined as “complexes emanating from the ventricles at a rate [of] >100 bpm” lasting for >30 s.^21^

### Group assignments

The medication treatment protocols at the University of Cincinnati Sarcoidosis Clinic have been described previously by Zhou et al.^6^ The patients’ immunosuppression and anti-arrhythmic medication regimens were classified into the following medication groups: no therapy, immunosuppression alone, anti-arrhythmic therapy alone, or dual therapy consisting of both immunosuppressive and anti-arrhythmic drugs. On review, there were various reasons as to why patients were not consistently on any medical therapy, including personal preferences, non-adherence, and/or adverse effects. In order for patients to be classified into a group, they had to have evidence of consistent medication orders and pharmacy dispenses over time. A subgroup analysis of patients on dual therapy who were taking either sotalol or amiodarone was performed to evaluate the impact of class III anti-arrhythmics.

### Statistical analysis

**Figure S1:**
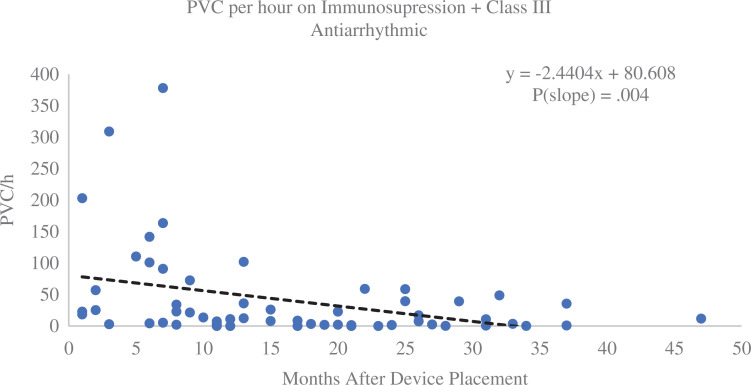
Premature ventricular contractions (PVCs) per hour for group 4b (immunosuppression + amiodarone or sotalol) over time. Patients on dual therapy that included amiodarone or sotalol experienced a marked decrease in PVCs over the course of the study.

**Figure S2:**
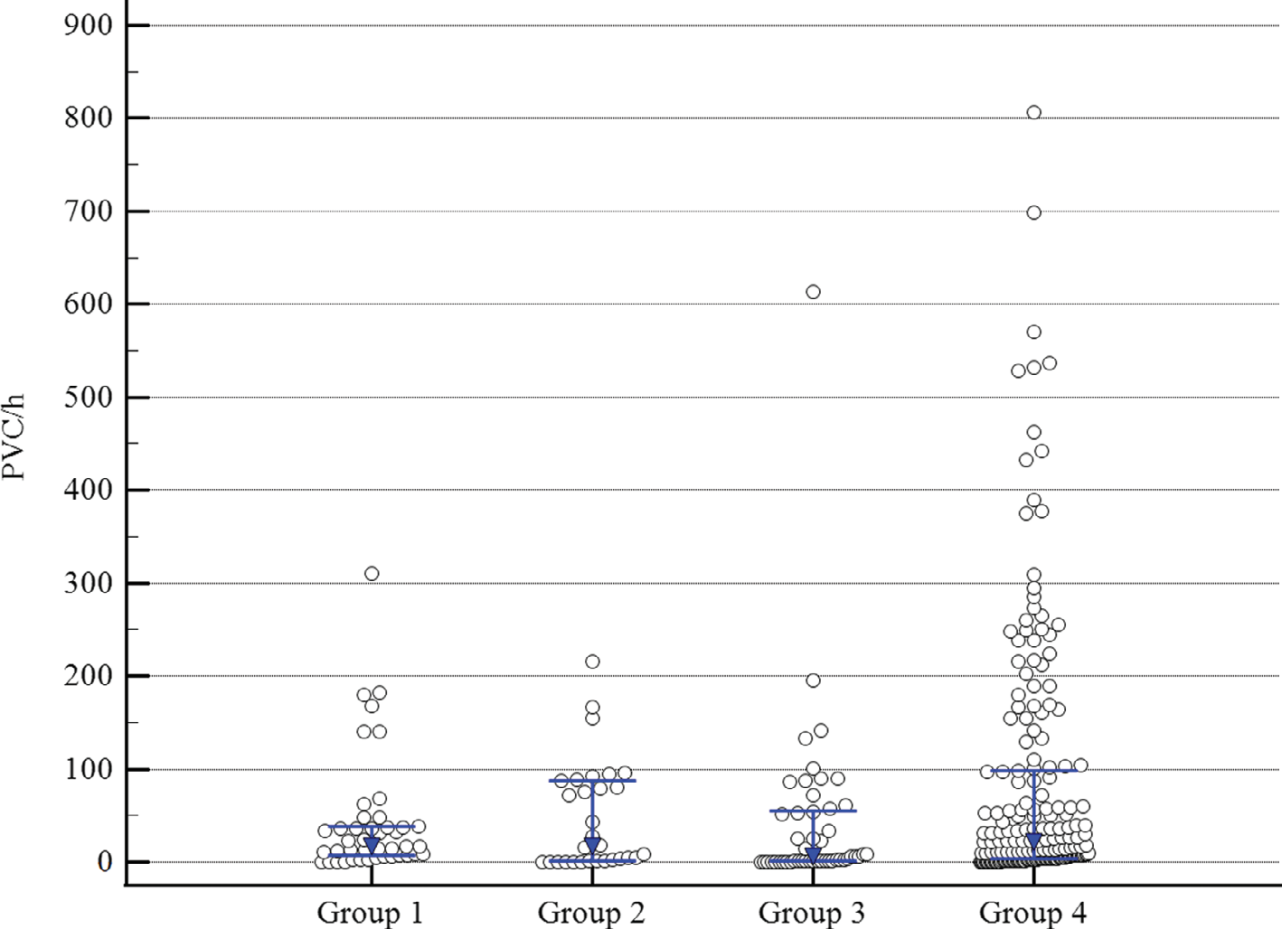
Premature ventricular contractions (PVCs) per hour for each interrogation over the follow-up period by treatment group. The blue markers and bars denote median and interquartile range (IQR) values, respectively. The PVC values for each group were as follows. Group 1 (no therapy) (n = 43): median, 17.3; IQR, 7.0–38.5. Group 2 (immunosuppression alone) (n = 30): median, 16.5; IQR, 1.4–87.3. Group 3 (anti-arrhythmic alone) (n = 49): median, 5.7; IQR, 0.9–54.8. Group 4 (dual therapy) (n = 199): median, 22.5; IQR, 3.5–101.8. Group 3 had a statistically lower median PVC count than group 4 (*P* = .035); otherwise, there were no differences between groups. *Abbreviations*: PVC/h, premature ventricular contractions per hour; n, number of PVC readings (1 per interrogation).

**Figure S3:**
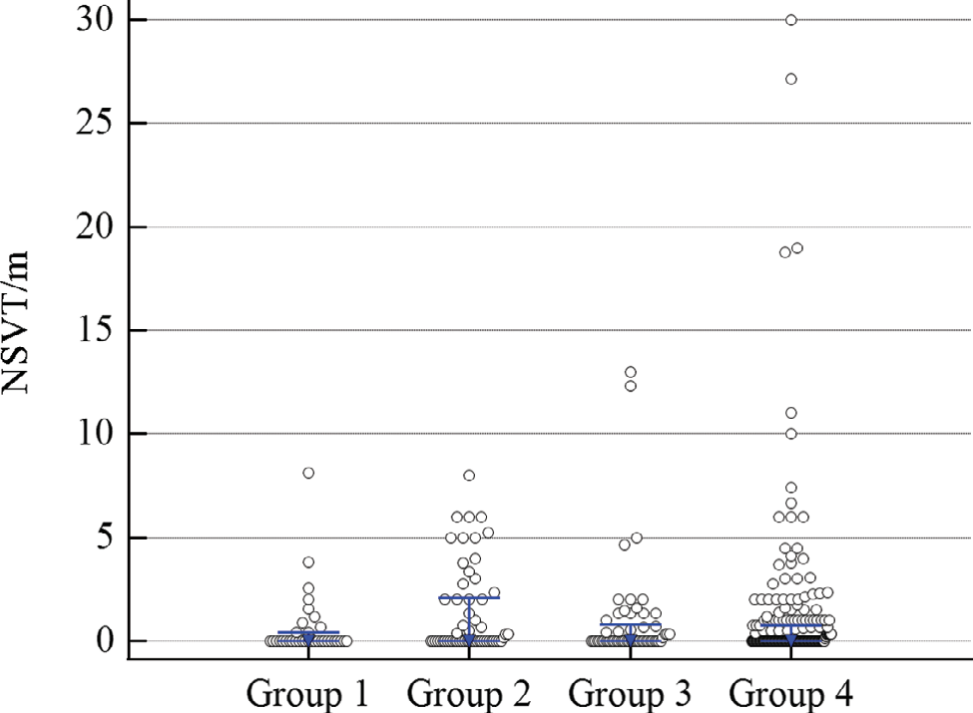
Average non-sustained ventricular tachycardia (NSVT) events per month from all interrogations over the follow-up period by treatment group. The blue markers and lines denote median and interquartile range (IQR) values, respectively. The NSVT values for each group were as follows. Group 1 (no therapy) n = 35: median, 0.0; IQR 0.0–0.43. Group 2 (immunosuppression alone) (n = 57): median, 0.0; IQR 0.0–2.08, Group 3 (anti-arrhythmic alone) (n = 57): median, 0.0; IQR, 0.0–0.68. Group 4 (dual therapy) (n = 246): median, 0; IQR, 0.0–1.0. *Abbreviation:* n, total number of NSVT values (1 value per interrogation).

**Figure S4:**
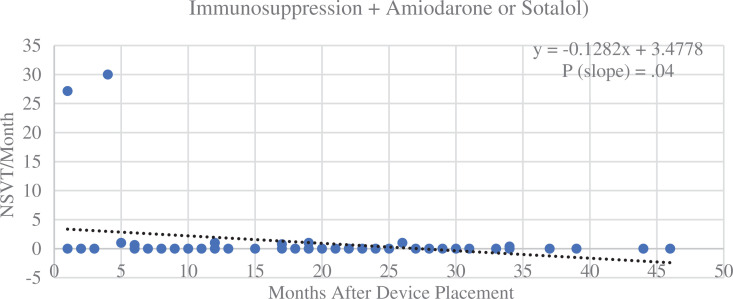
Non-sustained ventricular tachycardia (NSVT) episodes per month for patients on dual therapy, including amiodarone or sotalol, over time. There was a significant decrease in NSVT episodes over time in this subset of patients, with almost no episodes of NSVT occurring after 5 months of therapy.

The data were analyzed using MedCalc version 19.4 (MedCalc Software Ltd., Ostend, Belgium). The normality of distribution for PVC and NSVT data was calculated by the D’Agostino–Pearson test. The PVC and NSVT data were analyzed using regression, and the significance of the trendline x variable (slope) was calculated to determine the change over time. The median (interquartile range) PVC and NSVT values for each group were compared using the Kruskal–Wallis 1-way analysis of variance test; this analysis can be found in **Figures S1–S4** and **Tables S1–S3** (online). The rates of VT/VF and device therapy were compared between groups using chi-squared tests and reported as *P* values and χ^2^ values. Chi-squared tests were performed in Microsoft Excel for Mac, version 16.37 (Microsoft Corporation, Redmond, WA, USA). *P* < .05 was considered to be statistically significant.

## Results

### Baseline characteristics

From the initial registry of 210 patients, 42 were selected based on the study inclusion and exclusion criteria, as can be seen in **Figure 2**. The majority of the patients who were excluded did not have accessible device interrogations in their electronic medical record, although several patients had unclear medication regimens or possible alternate diagnoses. Three patients died during the course of the study, including 2 from progressive heart failure and 1 from metastatic cancer.

**Figure 1:**
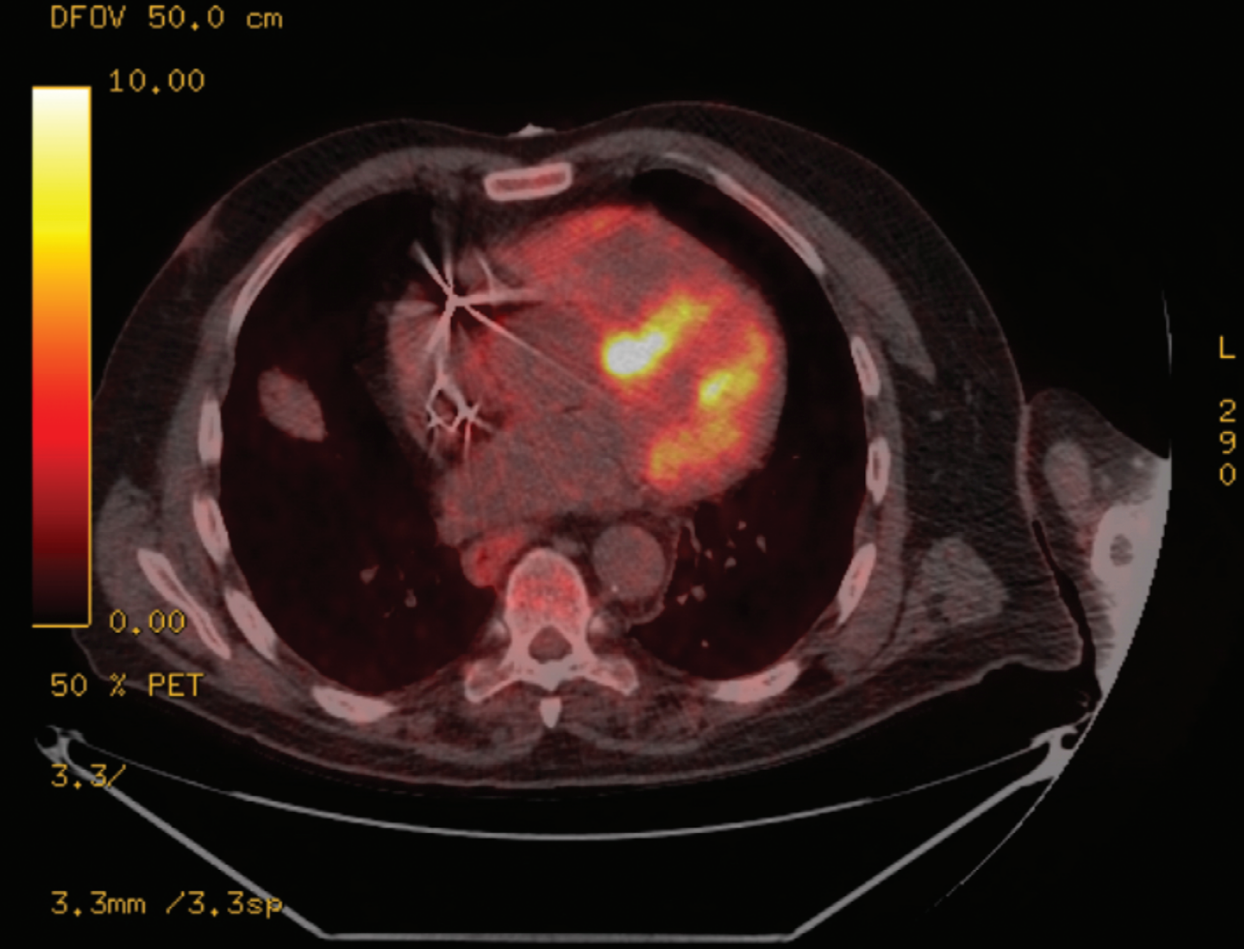
Fluorodeoxyglucose positron emission tomography myocardial metabolic evaluation for patient 42 showing focal uptake in the basal septum, left ventricular anterior wall, and (to a lesser extent) the free wall of the right ventricle, suggestive of active inflammation.

**Figure 2:**
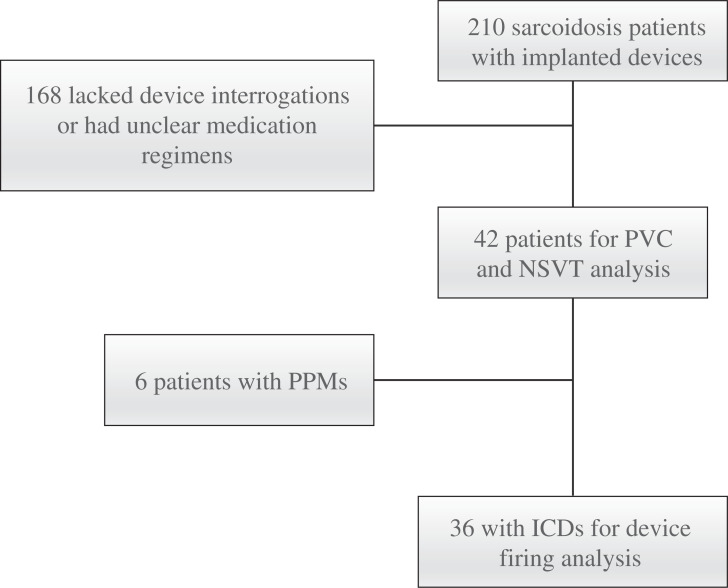
Patients with cardiac sarcoidosis reviewed for inclusion and exclusion. Device firing was defined as any indicated anti-tachycardia pacing or shock. *Abbreviations:* ICD, implantable cardioverter-defibrillator; NSVT, non-sustained ventricular tachycardia; PPM, permanent pacemaker; PVC, premature ventricular contraction.

The baseline characteristics are shown in **Table 1**. The mean age at device implantation was 55 years. Twenty-five subjects were Black and 17 were White, and 24 were women and 18 were men. The mean follow-up after device placement was 31 months, with a standard deviation of 13 months. The diagnosis of cardiac involvement and indications for the placement of a permanent pacemaker or an implantable cardioverter-defibrillator (ICD) are listed in **Table 1**. An example of the most frequently used imaging modality to support the diagnosis of CS can be found in **Figures 1** and **S1–S4** and **Tables S1–S3**. There were no significant differences between groups in demographics, cardiac systolic function, coronary artery disease, or indication for device placement. **Table 2** shows the specific immunosuppressive and anti-arrhythmic medications for patients in each group.

### Premature ventricular contraction and non-sustained ventricular tachycardia data

**Figure 3:**
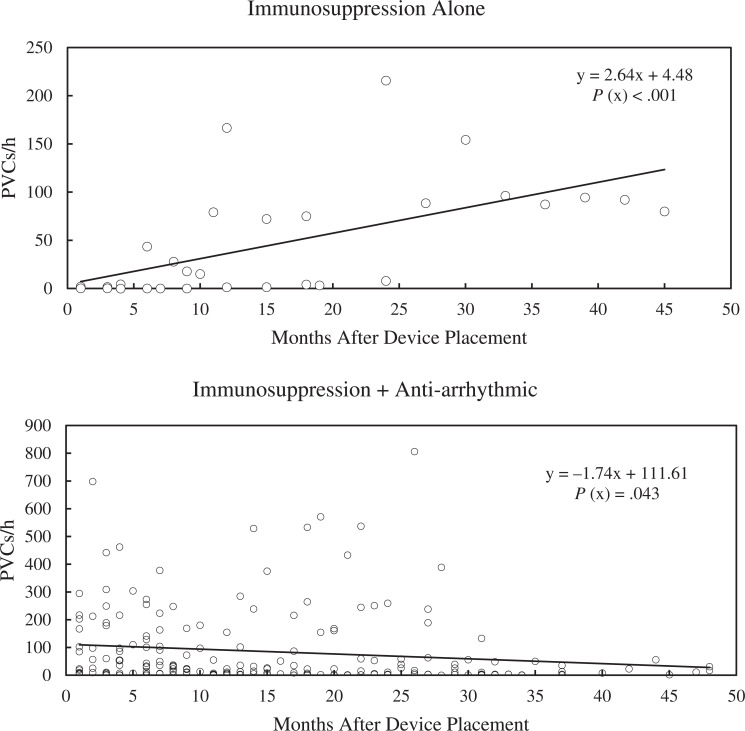
Premature ventricular complexes (PVCs) per hour over time for patients on immunosuppression alone compared to dual therapy. Patients on immunosuppression alone had a statistically significant increase in PVCs over time, whereas those on dual therapy had a decrease in PVCs over time. *P* values were calculated for the significance of the x variable (slope). The graphs for PVCs/h for the remaining treatment groups are provided in the Supplement. *Abbreviation:* PVC, premature ventricular contraction.

There were 398 interrogations with PVC and NSVT data available for review over a mean follow-up period of 31 (standard deviation, 13) months. Over the course of the study, the median PVC count for patients on anti-arrhythmics alone was lower than that for those on dual therapy; otherwise, there were no differences between groups. Patients on dual therapy had a higher starting PVC count than all other groups (median, 60 PVC/h; *P* < .001). The linear regression model for PVC versus time for the dual therapy group showed a significant decrease in PVCs over time (slope, −1.47; *R*^2^ = 0.02; *P* = .043), as shown in **Figure 3**. Patients taking immunosuppression alone had an increase in PVCs over time (slope, 2.64; *R*^2^ = 0.34; *P* < .001), as seen in **Figure 3**. There was an increase in PVCs seen in patients on no therapy and no change for patients on only anti-arrhythmics; graphs for these groups may be found in **Figures S1–S4**.

**Figure 4:**
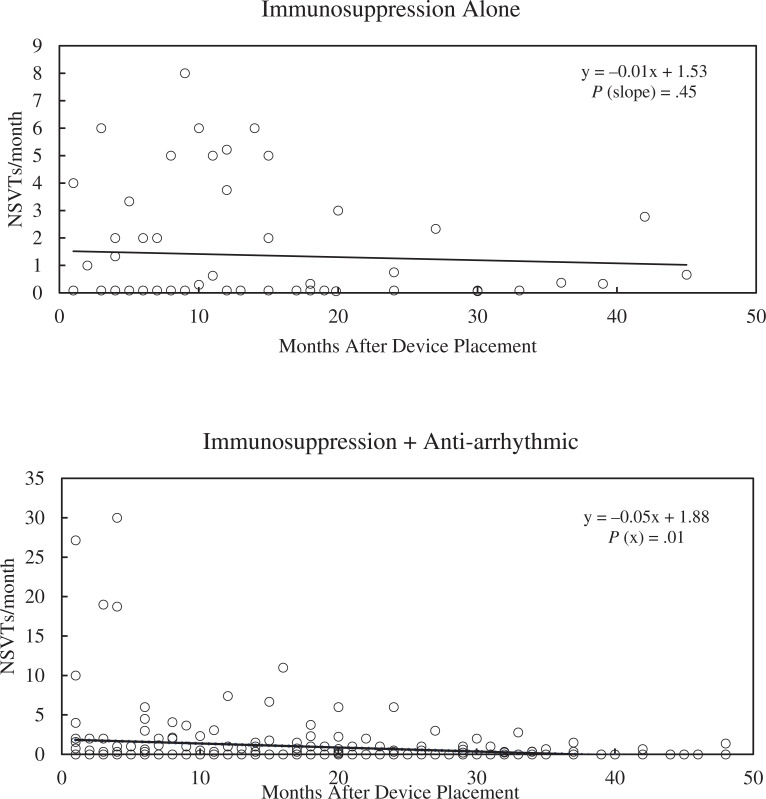
Non-sustained ventricular tachycardia (NSVT) episodes per month over time for patients on immunosuppression alone compared to dual therapy. The immunosuppression-alone patients had no significant change in the number of NSVT events over time, while those on dual therapy had a statistically significant decrease. The *P* value denotes significance of the x variable (slope). The graphs for NSVTs over time for the remaining treatment groups are provided in the Supplement. *Abbreviation:* NSVT, non-sustained ventricular tachycardia.

**Figure 5:**
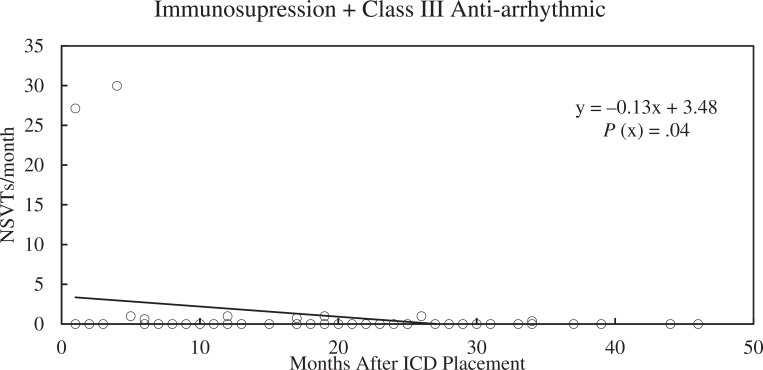
Non-sustained ventricular tachycardia (NSVT) episodes per month over time for a subgroup of patients on dual therapy whose regimen included a class III anti-arrhythmic (amiodarone or sotalol). Medical therapy in this group had a significant impact, with almost no NSVT events recorded after 5 months. The *P* value denotes significance of the x variable (slope). *Abbreviations:* ICD, implantable cardioverter-defibrillator; NSVT, non-sustained ventricular tachycardia.

There were no significant differences in the median number of NSVT events between groups over the course of the study. The dual therapy group had a significant decrease in NSVT over time (slope, −0.05; *R*^2^ = 0.03; *P* = .009), as shown in **Figure 4**. Patients on no therapy or single therapy did not see a significant change in NSVT frequency over time. A subgroup of patients on immunosuppression plus amiodarone or sotalol had a marked decrease in NSVT events (slope, −0.13; *R*^2^ = 0.08; *P* = .038), as seen in **Figure 5**.

### Ventricular tachycardia/ventricular fibrillation and device firings

There were 51 appropriate device therapies for 31 episodes of VT and 20 episodes of VF during the course of the study. Device firings (ATP or shock) occurred in 10 of 36 (28%) patients with ICDs, and most of them (n = 10) had multiple arrhythmias/device therapies. Two patients had arrhythmias that were refractory to medical therapy and underwent ablation, including 1 with VT storm. There was no significant difference in the percentage of patients experiencing device firings according to the medication regimen, as shown in **Table 3**. A subgroup analysis was performed of patients without prior VT/VF who had ICDs placed for primary prevention. In this subgroup, dual therapy resulted in a significantly lower rate of device firings (6%) than single (43%) or no therapy (40%) (*P* = .049, χ^2^ = 6.02), as can be seen in **Table 3**.

## Discussion

In this study, the impact of corticosteroid-sparing immunosuppression with and without anti-arrhythmic therapy was assessed in patients with CS. To the best of our knowledge, this is the first report comparing corticosteroid-sparing immunosuppression with anti-arrhythmics to immunosuppression alone for the treatment of arrhythmias in CS. Patients on dual therapy experienced a reduction in PVCs and NSVT events over time, whereas the other treatment groups showed no improvement. Patients on dual therapy did have higher burdens of PVC and NSVT to begin with, which was likely due to selection bias.

Overall, there was a high frequency of device firings in the patients studied, and there were no differences across medication groups in rates of device therapy. An interesting finding was that a subset of patients who had received ICDs for the primary prevention of sudden cardiac death required fewer device firings when on dual medical therapy compared to single or no therapy. This subset analysis may warrant further investigation in future studies but should not detract from the main finding of no statistical difference based on the medication regimen.

The current guidelines to treat arrhythmias recommend immunosuppression initially, followed by anti-arrhythmics for refractory arrhythmias.^15^ The results of this study suggest that starting with both medication classes could be more effective; however, further research would be needed to determine if this is truly the case. There has been a proposed update that anti-arrhythmics should be considered in CS patients with a history of ventricular arrhythmias even if they are already on immunosuppression.^22^ A prospective, randomized study of CS patients with implanted devices tracked in a similar manner could be helpful to conclude if this update would have a positive clinical impact.

### Effects of medical therapy on ventricular ectopy

Device interrogations revealed a high degree of variability in the PVC count from patient to patient over the course of the study. This was not unexpected given the variability in PVC frequency in the general population,^23^ combined with clinical differences in the study patients. Because of this, PVCs were graphed over time to show the change from baseline with therapy during the follow-up period. Notably, patients on dual therapy experienced a decrease in PVC count over time, while those on immunosuppression alone had increasing PVC counts. The decrease in PVCs could have been clinically significant for patients with very high PVC burdens or those showing symptoms from their PVCs. Several patients in the study had high enough burdens to be at risk for PVC-induced heart failure and therefore would have benefited from suppresion.^20,24,25^ However, as PVC treatment does not have a significant effect on outcomes,^20^ the need for medical therapy in these patients should not be determined by PVC count and would have to be indicated based on the clinical assessment of cardiac function.

The results showed a reduction in NSVT over time with dual therapy, which was not seen in the other groups. While the negative slope of the NSVT trendline for dual therapy was statistically significant, the magnitude of change was low and not likely to be of clinical importance. Of note, patients on immunosuppression with amiodarone or sotalol had a profound decrease in NSVT, with almost no recurrence after several months of therapy. The class III anti-arrhythmics were likely the main drivers of this, as those on β-blockers and immunosuppression did not see such a decrease. In terms of clinical use, the potential benefits of NSVT reduction would need to be balanced with the risk of adverse effects from the anti-arrhythmic medications.

### Sustained ventricular arrhythmias and device firing

The frequency of VT/VF in our CS patients (28%) was similar to what has been reported previously—that is, 21% over a 4.1-year follow-up period in 1 study and 38% over 2.6 years in another.^9,13^ Most patients with prior VT/VF experienced arrhythmia recurrence (71%) despite dual medical therapy. Difficulty in preventing arrhythmia recurrence in CS patients has been described in prior reports.^13,26^ Arrhythmias can be difficult to control as they are caused by macro–re-entry around scarred myocardium, which is highly arrhythmogenic and not amenable to immunosuppression.^9,15,27^ The recurrence rate of VT in patients on medical therapy in this study (71%) was higher than rates given in previous reports, such as 38% in a study with 37 patients and 57% in another with 21 patients.^16,11^ All of the patients in our study had ICDs, which may have led to the higher detection rate. Despite the high rate of arrhythmia recurrence in patients with prior VT/VF, there was an improvement in the arrhythmia frequency with treatment over time. Patient 22, for example, had 7 episodes of VF after device implantation, but none after 3 months of methotrexate and amiodarone. Patient 32 had a VT storm with 17 device therapies, underwent ablation, and was maintained on medical therapy without relapse thereafter.

Patients with prior VT/VF were predominantly on dual therapy. The high rates of arrhythmia recurrence in these patients influenced the comparison of device firings between groups. In patients without prior VT/VF (who had devices placed for primary prevention), there were significantly fewer device firings when patients were on dual medical therapy. In these patients, anti-inflammatories may have reduced lymphocytic infiltration and scar formation, while anti-arrhythmics would have suppressed rhythms originating from the pre-existing scar.

### Limitations and future investigations

There were several limitations to this study due to its retrospective, non-randomized design. Device implantation was not aligned with the initiation of medical therapy in many of the patients; therefore, before- and after-medication arrhythmia counts were not able to be determined. Selection bias was present, in that patients on maximal medical therapy had higher PVC counts to begin with and therefore had greater room for improvement with therapy. There was an uneven distribution of patients in the treatment groups, with fewer patients on no therapy or single-agent therapy compared to dual therapy. The low number of patients in these groups limited the power to detect differences in arrhythmias and device therapy between groups. There was also significant heterogeneity in the specific medication regimens patients were taking, which limits the generalizability of these findings to individual patients. Lastly, there was a high-degree variability in PVC and NSVT counts from patient to patient, which made it difficult to assess for trends in these data over time and to compare the results between groups. In the future, a prospective, randomized study design with patients assigned to predetermined medication regimens would resolve many of these issues and allow for a more accurate assessment of the impact of medical therapy.

## Conclusion

This study was performed to investigate the effectiveness of medical management for arrhythmias in CS patients with implanted devices. We found that patients on dual therapy with both immunosuppression and anti-arrhythmics had a reduction in PVCs and NSVT, while patients on single or no therapy did not. There were no differences between groups in terms of device firings across all patients; however, in patients with devices placed for primary prevention, dual therapy led to fewer device firings throughout the study. A prospective study with assigned medication regimens and scheduled device interrogations would be ideal to further investigate the optimal medical management of arrhythmias in these patients. Such a study would likely have to be multicentered to have a sufficient number of patients in each treatment group.

## Figures and Tables

**Table 1:** Baseline Characteristics, Diagnoses, and Device Indications According to Medication Group

	No Therapy n = 7	Immunosuppressive n = 4	Anti-arrhythmic n = 8	Dual Therapy n = 23	Total n = 42
Mean age at device placement (years)	61.3	47.8	57.9	55	55
Age range (years)	37–79	41–62	38–76	29–67	29–79
Race, Black/White	6/1	1/3	5/3	13/10	25/17
Sex, female/male	3/4	3/1	5/3	13/10	24/18
Mean LVEF	41%	59%	50%	44%	47%
Obstructive CAD	0%	0%	0%	17%	9.5%
Mean follow-up (months)	35.3	36.6	21.0	31.7	31
Standard deviation follow-up	8.8	11.9	12.7	12.8	12.7
Sarcoidosis diagnosis					
Lung biopsy	3	2	3	10	18
Mediastinal lymph node biopsy	1	1	2	3	7
Cardiac biopsy	0	0	0	1	1
Other biopsy^*^	3	1	3	9	16
Cardiac involvement diagnosis					
Positive MRI	0	2	4	8	14
Positive PET	3	2	3	8	16
Clinical diagnosis^†^	4	0	1	6	11
Indication for PPM/ICD					
Heart block	5	2	3	4	14
VT/VF	0	0	1	6	8
HFrEF	1	1	3	5	10
Other indication^‡^	1	1	1	8	10

*Abbreviations:* CAD, coronary artery disease; HFrEF, heart failure with reduced ejection fraction; ICD, implantable cardioverter-defibrillator; LVEF, left ventricular ejection fraction; MRI, magnetic resonance imaging; PET, positron emission tomography; PPM, permanent pacemaker; VT/VF, ventricular tachycardia/ventricular fibrillation. ^*^Other biopsy includes biopsy of the skin, peripheral lymph nodes, or bone marrow. ^†^Clinical diagnosis includes unexplained reduced left ventricular ejection fraction, complete heart block, or response to immunosuppression. ^‡^Other indications include inducible VT, frequent ectopy with syncope, and family history of sudden cardiac death.

**Table 2:** Specific Medication Regimens by Group

	No Therapy n = 7	Immunosuppressive n = 4	Anti-arrhythmic n = 8	Dual Therapy n = 18	Total n = 42
Immunosuppression					
Methotrexate regimen^*^	0	3	0	14	17
Azathioprine regimen^†^	0	1	0	5	6
Prednisone alone	0	0	0	2	2
Other^‡^	0	0	0	2	2
Anti-arrhythmic					
Metoprolol	0	0	6	10	16
Carvedilol	0	0	2	11	13
Amiodarone	0	0	0	4	4
Sotalol	0	0	0	1	1

^*^Methotrexate regimen includes methotrexate ± prednisone or hydroxychloroquine. ^†^Azathioprine regimen includes azathioprine ± prednisone or hydroxychloroquine. ^‡^Other regimens include infliximab + prednisone and mycophenolate + prednisone.

**Table 3:** Percent of Patients with Implantable Cardioverter-defibrillators Experiencing Device Firing over the Study Time Frame

All Patients with ICDs	Device Firing	No Device Firing	Percent
No therapy	2	3	40%
Single therapy	3	4	43%
Dual therapy	5	18	22%
**ICDs for Primary Prevention**			
No therapy	2	3	40%
Single therapy	3	4	43%
Dual therapy	1	16	6%

*Abbreviation:* ICD, implantable cardioverter-defibrillator. Single therapy included patients on either immunosuppression alone or anti-arrhythmic medication alone. Dual therapy included patients on both immunosuppression and an anti-arrhythmic. Device firings included anti-tachycardia pacing or device shock for a sustained ventricular arrhythmia. For all patients with ICDs: *P* = 0.35, χ^2^ = 3.31. For patients with devices placed for primary prevention: *P* = 0.04, χ^2^ = 3.31.

**Table S1:** Kruskal–Wallis Analysis of Variance of Premature Ventricular Contractions per Hour for Groups 1–4

Kruskal–Wallis Test	
Test statistic	8.5933
H value corrected for ties (t)	8.5942
Degrees of freedom	3
Significance level	*P* = .035203

Post Hoc Analysis (Conover)			
Group	N	Average Rank	Difference (*P* < .05) from Factor nr
(1) No therapy	43	163.99	
(2) Immunosuppression	30	150.08	
(3) Anti-arrhythmic	49	127.97	(4)
(4) Dual Therapy	199	170.13	(3)

As can be seen, group 3 had a statistically lower median premature ventricular contractions per hour value than group 4 (*P* = .0352).

**Table S2:** Kruskal–Wallis Analysis of Variance of Non-sustained Ventricular Tachycardia Events per Month for Groups 1–4

Kruskal–Wallis Test	
Test statistic	3.5596
H value corrected for ties (t)	4.5904
Degrees of freedom (DF)	3
Significance level	*P* = 0.204364

Group	N	Average Rank
No therapy	35	176.57
Immunosuppression	57	220.69
Anti-arrhythmic	57	196.24
Dual therapy	246	196.20

There was no significant difference in the median number of non-sustained ventricular tachycardia events between the 4 groups.

**Table S3:** Percent of Patients Experiencing Device Firing by Implantable Cardioverter-defibrillator Indication

Indication	Device Firing	No Device Firing	Percent
VT/VF	5	2	71%
HFrEF	4	5	44%
Conduction block	2	8	20%
Other*	0	10	0%

*Abbreviations:* HFrEF, heart failure with reduced ejection fraction (<40%); ICD, implantable cardioverter-defibrillator; VF, ventricular fibrillation; VT, ventricular tachycardia. Device firing refers to anti-tachycardia pacing or shock for VT/VF. Other refers to cardiac sarcoidosis with either syncope (n = bib2), inducible VT (n = bib4), family history of sudden cardiac death (n = bib1), or high ectopy burden (n = bib3). *P* = .01, χ^2^ = 11.25.
